# Quantifying Environmental Limiting Factors on Tree Cover Using Geospatial Data

**DOI:** 10.1371/journal.pone.0114648

**Published:** 2015-02-18

**Authors:** Jonathan A. Greenberg, Maria J. Santos, Solomon Z. Dobrowski, Vern C. Vanderbilt, Susan L. Ustin

**Affiliations:** 1 Department of Geography and Geographic Information Science, University of Illinois at Urbana-Champaign, Champaign, Illinois, United States of America; 2 Department of Innovation, Environmental and Energy Sciences, Utrecht University, Utrecht, The Netherlands; 3 Department of Forest Management, College of Forestry and Conservation, University of Montana, Missoula, Montana, United States of America; 4 NASA Ames Research Center, Mountain View, California, United States of America; 5 Center for Spatial Technologies and Remote Sensing (CSTARS), Department of Land, Air and Water Resources, University of California Davis, Davis, California, United States of America; Albert-Ludwigs-Universitat Freiburg, GERMANY

## Abstract

Environmental limiting factors (ELFs) are the thresholds that determine the maximum or minimum biological response for a given suite of environmental conditions. We asked the following questions: 1) Can we detect ELFs on percent tree cover across the eastern slopes of the Lake Tahoe Basin, NV? 2) How are the ELFs distributed spatially? 3) To what extent are unmeasured environmental factors limiting tree cover? ELFs are difficult to quantify as they require significant sample sizes. We addressed this by using geospatial data over a relatively large spatial extent, where the wall-to-wall sampling ensures the inclusion of rare data points which define the minimum or maximum response to environmental factors. We tested mean temperature, minimum temperature, potential evapotranspiration (PET) and PET minus precipitation (PET-P) as potential limiting factors on percent tree cover. We found that the study area showed system-wide limitations on tree cover, and each of the factors showed evidence of being limiting on tree cover. However, only 1.2% of the total area appeared to be limited by the four (4) environmental factors, suggesting other unmeasured factors are limiting much of the tree cover in the study area. Where sites were near their theoretical maximum, non-forest sites (tree cover < 25%) were primarily limited by cold mean temperatures, open-canopy forest sites (tree cover between 25% and 60%) were primarily limited by evaporative demand, and closed-canopy forests were not limited by any particular environmental factor. The detection of ELFs is necessary in order to fully understand the width of limitations that species experience within their geographic range.

## Introduction

Environmental limiting factors (ELFs) are thresholds that determine the maximum or minimum biological response for a given suite of environmental conditions. This biological response conditions species and ecosystem geographic distributions, and thus environmental limiting factors determine and are determined by organisms’ life-history traits, and are found in many aspects of ecology such as niche theory [1], physiological ecology [2,3], and others. While limiting the spatial distribution of a species or ecosystem, ELFs are more than factors that determine ecotones (sensu [4]). ELFs can determine the response thresholds of biological responses such as abundance and density [5, 6], cover [7], growth rate [8], biomass [9], etc. For example in forestry, the definition of “site quality” is an ELF in that it quantifies the maximum forest stand response (e.g. stand density, percent tree cover) under a given site’s environmental conditions [7]. In coupled ecosystem-climate models, environmental limiting factors define, for instance, asymptotes in growth curves under a given suite of environmental conditions and threshold responses to extreme environmental conditions [10].

ELFs, at least in theory, can be used to elucidate mechanistic/physiological tolerances of an organism. Empirical approaches have been used to estimate these tolerances. Laboratory studies have investigated tolerances to varying climate conditions [11]. Growth studies have been performed in botanical gardens to determine ELFs [12]. In the field, Littell et al. [13] showed how water limits Douglas fir (*Pseudotsuga menziesii*) growth, and Rehfeldt et al. [8] performed an empirical analysis of plant growth limitations associated with climate. However, the determination of ELFs may be limited by the number of samples used in such empirical analyses [8,13]. When scaling to the landscape level or the geographical range of a species or ecosystem, empirical approaches may become impractical. Further, because ELFs determine thresholds they are inherently ‘rare’, identifying them requires large sample sizes and specific techniques to detect them.

**Fig 1:**
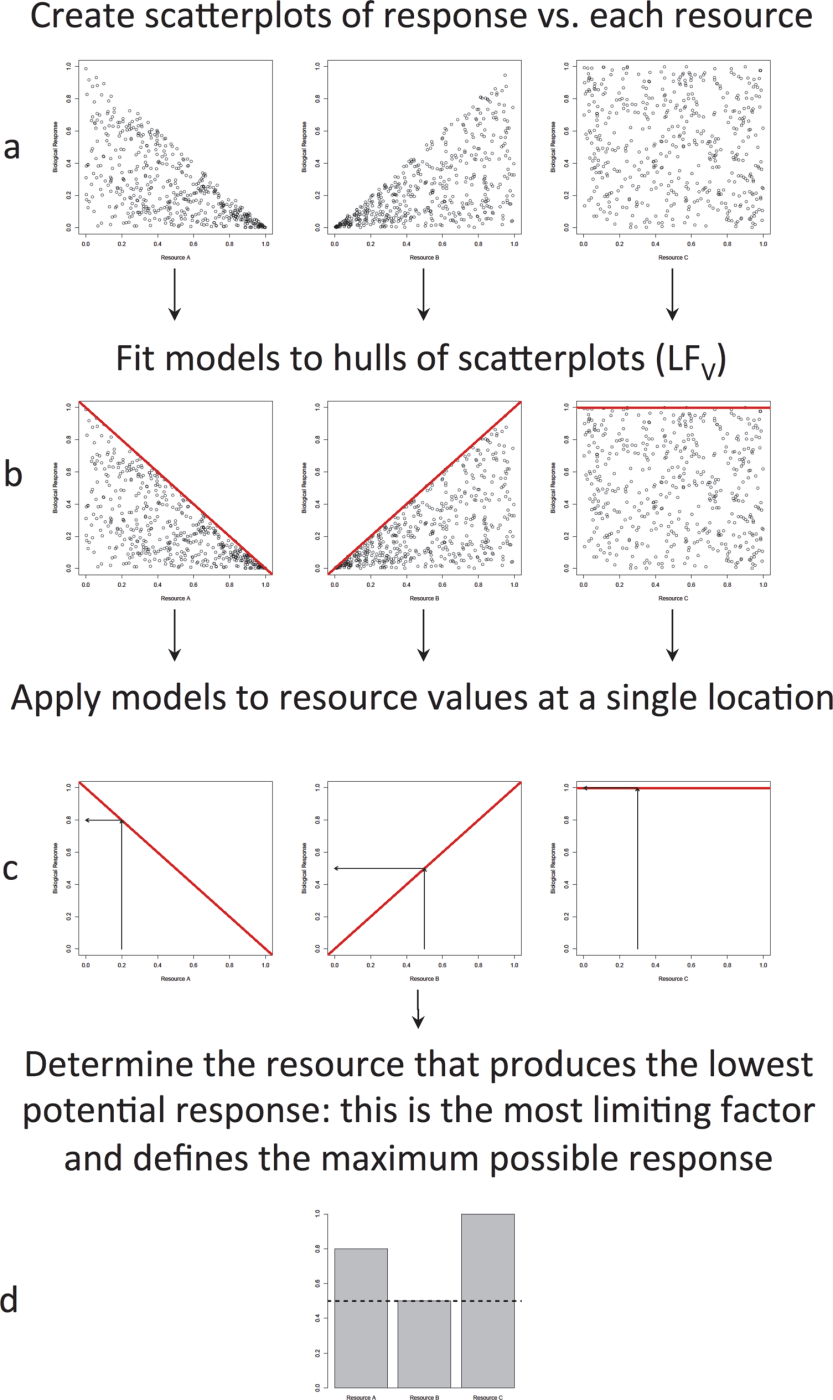
Conceptual figure on how to determine an ELF using the principle of limiting factors. a) Biological response data is plotted against potentially limiting resources (adapted from Fig. 3 in Cade and Noon [16]). b) Boundary models (*LF*_*V*_) are fit to the upper hull of each scatterplot. c) To produce a prediction of the maximum biological response as well as the identity of the most limiting factor at a given site with a set of measured resource values, the *LF*_*V*_ models are applied to these resource values, and d) the resource that predicts the lowest maximum biological response is chosen as the most limiting factor, and its prediction is that site’s maximum biological response.

Formally, ELFs correspond to the upper and lower quantiles of the distribution of responses of an organism to a suite of environmental conditions, and modifications of traditional correlational analysis have been used to describe these responses. For example, Cade et al. [9] demonstrated the usefulness of techniques such as quantile regression to estimate the effect of canopy cover on oak (*Quercus* spp.) acorn biomass, and lily (*Erythronium grandiflorum*) numbers as a function of limiting factors. Cade and Guo [14] showed how quantile regression could be useful to estimate the limiting factors to summer densities of desert plants. The advantage of these techniques is that they account for the principle of limiting factors. The principle of limiting factors states that there is one (and only one) environmental condition that limits a response at any given point and time within a distribution range (originally described in Sprengel [15], but for a modern description see [16]). Scatterplots between a biological response variable and a potential limiting factor often yield “triangular distributions” (Fig. 1A, after [17]), where a well-defined hull is present but many samples are still below this hull (or the 99% quantile regression line). These patterns are likely indicative of multivariate constraints on the biological response [16]. Individual samples deviating from the estimated hulls are interpreted not as a poor model fit, but as indications of additional, unmeasured limiting factors [16].

Although ELFs abound as theoretical constructs across many aspects of ecology, as do tools to analyze these limiting factors (e.g. quantile regression [16, 18]), quantifying these conditions remains problematic. The detection of boundaries requires significantly more data than central tendency estimations, as boundaries tend to be data-poor (i.e. there are typically less examples of a maximum or minimum response than an average response). The sample size problem is further exacerbated by the typically multivariate nature of ELFs (e.g. a plant can be limited by a suite of bioclimatic factors, including temperature, light, and water; [19]), which can significantly increase the sample size requirements. To solve this issue either concentrated sampling around suspected boundaries is required, or significantly larger datasets are needed. Given the wide availability of spatially continuous remotely sensed estimates of biological variables (for example, tree cover [20], tree density [21]) and climate [22]) a tractable approach to deriving ELFs for such responses becomes possible.

In this analysis, we focus on defining the multivariate ELFs of percent tree cover in eastern Lake Tahoe Basin, NV, a region which qualitatively showed evidence of both significant water balance and temperature limitations [21]. We extend the original work described by Greenberg et al. [21] by using actual climate surfaces instead of the topographically derived environmental gradients (elevation and potential relative radiation, MWh/m^2^/year) used before, to address the following questions: (1) can we detect environmental limiting factors on percent tree cover in the Lake Tahoe Basin, NV, and, if so, what do these conditions appear to be? (2) how are the environmental limiting factors distributed spatially, and (3) to what extent are unmeasured environmental factors limiting tree cover? We selected percent tree cover as the biological response variable because it is, essentially, the per-area probability that a tree can establish, grow to a detectable size, and avoid mortality. Thus, it is a fundamental variable that ties physiological response to environmental conditions.

## Methods

### Study area

**Fig 2:**
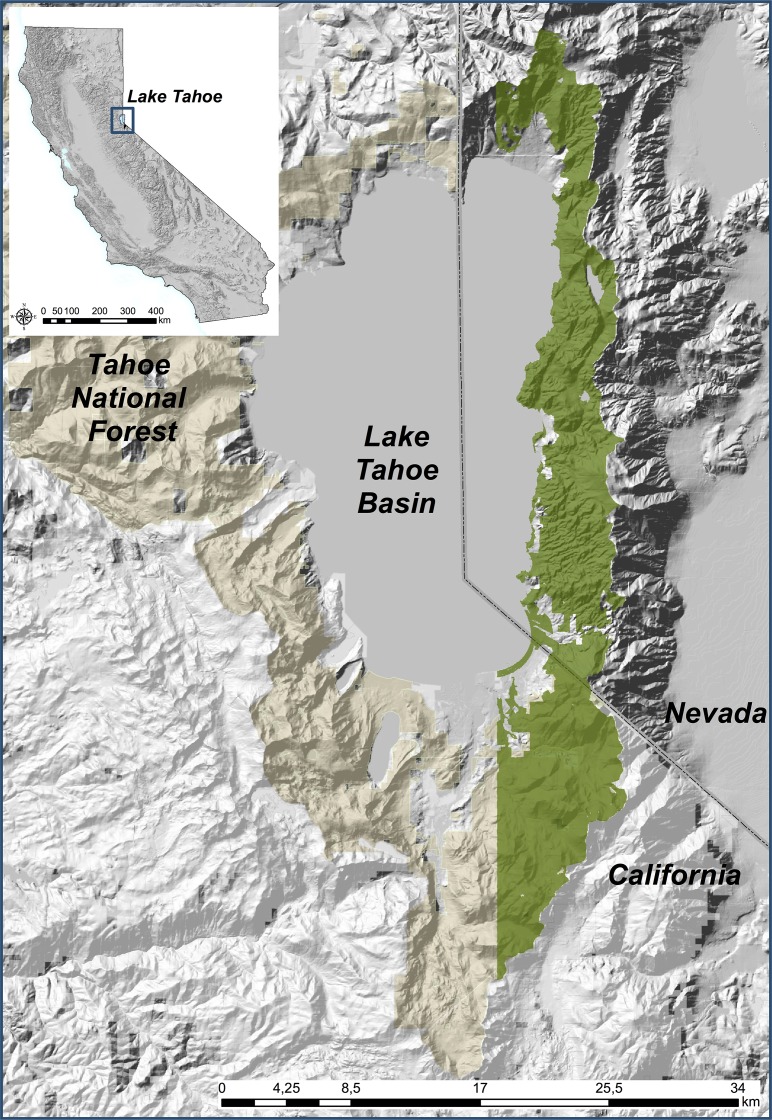
The study area for this analysis are the eastern slopes of the Lake Tahoe Basin, CA/NV.

Our study area covers 26,730 ha of the eastern shore of the Lake Tahoe Basin, NV (Fig. 2). The elevation ranges from 1900m above sea level (asl) at the lakeshore to 3400m asl. Most of the Basin is dominated by forests (67% of the study area), with Jeffrey pine (*Pinus jeffreyi*), white fir (*Abies concolor*) and red fir (*Abies magnifica*) dominated forests being the most common communities [21]. Average daily temperature ranges between -2°C in the winter and 13°C in the summer, and average annual precipitation is 786mm, in the form of rain or snow. Two-thirds of the forest experienced logging during the 19^th^ century [21]. This study area encompasses the entire area acquired at nadir by the 2002 Lake Tahoe Basin IKONOS satellite data acquisition.

### Remote sensing derived tree cover

In Greenberg et al. [21], we described a technique by which we were able to accurately map individual trees using hyperspatial imagery (pixel sizes smaller than an object of interest). This technique allowed for accurate tree presence/absence and per-tree crown area to be calculated, with a cross-correlation between modeled tree presence/absence and photointerpreted tree presence/absence of 0.8773. The technique was developed with an IKONOS flightline collected on 19 July 2002. IKONOS collects multispectral data with 4m spatial resolution, and panchromatic data with 1m spatial resolution. The imagery was pan-sharpened to create a 4-band image with 1m pixels. Estimated pre-tree crown areas were summarized at 30m resolution to produce a surface of percent tree cover (hereafter “tree cover”). This scale was chosen to best match the scale of the environmental surfaces, which were derived from a 30m DEM. These data represent the biological response variable to be used in this analysis.

### Environmental surfaces: bioclimatic variables

To assess whether ELFs exist on tree cover, we chose predictor variables based on three factors: a) the variables were climate surfaces, rather than topographic surrogates (e.g. slope, aspect, elevation, topographic convergence, [24]), and were at a sufficient resolution to capture the microclimate factors at play in this ecosystem, b) the surfaces were biologically meaningful, i.e., climate variables that have a known relation with the biological response variable (sensu [22]), and c) they all had correlation coefficients less than 0.8 (e.g. they were not highly collinear). This is a conservative correlation coefficient, in comparison to the value proposed by Dormann et al. [25], but we wanted to ensure that the resulting ELFs were a function of a single limiting factor.

Plant growth is limited by energy and water availability, each of which can affect growth separately or by interaction. For example, Zeng et al. [26] showed how maximum tree cover in tropical areas occurs at intermediate temperature and precipitation values. The following bioclimate surfaces were chosen for use in our analysis: annual average temperature (°C), annual average minimum temperature (°C), annual potential evapotranspiration (“PET”, mm), and annual water balance (PET minus precipitation, “PET-P”, mm). These variables were selected to include temperature effects (average and minimum), and interactions between temperature and water availability, as measured by potential evapotranspiration and PET-P. These base bioclimate summary surfaces were derived from monthly temperature and precipitation estimates using topographically mediated downscaling as described in Dobrowski et al. [23], monthly clear-sky radiation estimates using the r.sun algorithm [27] and monthly wind speed modeling using WindWizard [28]. Potential evapotranspiration was derived from these base surfaces following Allen et al. [29] and water balance by subtracting annual precipitation from PET. All surfaces were produced at a 30m ground sample distance (GSD) to match the percent tree cover product.

### Fused tree cover-bioclimate database

We fused the vegetation response surfaces (percent tree cover) with the bioclimate predictor surfaces by creating a layer stack, and extracted all pixel values falling within a mask created by the intersection of Lake Tahoe Basin Management Unit (LTBMU) boundary, the IKONOS flightline, a map of terrestrial cover with all water features removed, and a map of non-urban regions. The final database contained 297,060 “plots” with a GSD of 30m.

Because of the characteristic wall-to-wall sampling of the remote sensing derived surfaces there is a potential for spatial autocorrelation to be present. Nonetheless, spatial autocorrelation, the property that less distant samples are more likely to be similar than farther away samples, is only a problem when using methods that require the assumption of independence or random selection of samples, to meet central limit theorem requirements for sampling design and analytical methods. As above mentioned ELFs do not comply to central limit theorem restrictions as they are the extremes or the “rare” events in a distribution, thus we believe that in our case subsampling the data to reduce spatial autocorrelation would only reduce the already scarce amount of information to detect ELFs.

### Limiting factor analysis

We assume the principle of limiting factors: that one, and only one, factor (in our case a bioclimatic variable) will control maximum tree cover at a given location and time. This assumption allowed us to analyze each bioclimatic variable independently, despite the fact that bioclimatic variables can be potentially inter-linked and correlated. Our goal is to derive and analyze a limiting factor model for a set of measured bioclimatic variables (*LF*_*measured*_) such that for a set of *N* bioclimatic variables *{V*_*1*_,*V*_*2*_,*…*,*V*_*N*_ *}* (in our case, mean temperature, minimum temperature, PET, and PET-P) we can determine the maximum biological response (tree cover) across all measured factors (*B*_*max*,*measured*_) as well as the identity of the limiting bioclimatic variable (*B*_*V*,*measured*_):

$$\left\{B_{max,measured},\;B_{V,measured}\}\;=\;{LF}_{measured}(V1,V2,\ldots ,VN\right)$$

Fig. 1 details the steps of this analysis. *LF*_*measured*_ is constructed by first deriving a per-variable limiting factor model *LF*_*Vi*_ which predicts the maximum biological response as a function of a single factor *V*_*i*_:

$$B_{max,Vi}\;=\;{LF}_{Vi}(Vi)$$

There are several ways to construct the function *LF*_*V*_ (e.g. quantile regression [18]). For our analysis, we subdivided the bioclimatic variable values into equal sized bins (25 bins per variable, chosen to insure a sufficient amount of plots per bin while not overfitting the data) ranging from the minimum to the maximum of the range of that bioclimatic variable exhibited across the entire study area. For each bin, we selected all the plots that fell into that bin, and calculated the maximum biological response (tree cover) for those plots. Thus, *LF*_*V*_ was effectively a lookup table with 25 possible values.

For *N* measured factors, there will be *N LF*_*Vi*_ models. Implementing the principle of limiting factors, we can construct the model *LF*_*measured*_ as the model that produces the minimum biological response:

$${LF}_{measured}\;=\;min({LF}_{V1}(V_{1}),\;{LF}_{V2}(V_{2}),\ldots ,\;{LF}_{VN}(V_{N}))\;$$

To produce a spatially explicit map of *B*_*max*,*measured*_ and *B*_*V*,*measured*_, the model *LF*_*measured*_ was applied to the set of bioclimatic predictor surfaces.

### Forest Type Classes

To assist in summarizing the results, we followed the FGDC 1997 [35] vegetation standards for forest type classes, we classified each location within our study area based on the actual tree cover and the maximum tree cover predicted by the model: ≥ 60% potential tree cover was classified as “Closed Tree Canopy”, between 25% and 60% tree cover as “Open Tree Canopy”, and less than 25% tree cover as “Non-Forest”.

### Model accuracy

There is no independent validation data available for this analysis, so we used a bootstrapping approach to determine the accuracy of the limiting factor model *LF*_*measured*_. Using the tree cover-bioclimate dataset, we created N = 500 limiting factor models by sampling, with replacement, from the input tree cover-bioclimate dataset. These models were applied to the original input dataset, producing N = 500 estimates of the per-variable limiting models *LF*_*Vi*_, the maximum biological response *B*_*max*,*measured*_ and the identity of the limiting factor *B*_*V*,*measured*_. From these N = 500 estimates, we used an empirical distribution function to calculate the mean and 95% confidence intervals around the models *LF*_*Vi*_, the system-wide mean and standard deviation of *B*_*max*,*measured*_, and the frequency of bootstrapped replicates that predict *B*_*V*,*measured*_. The frequency of the bootstrapped replicates indicates the stability of the prediction, and will range between 1/N (low stability, where N = the number of bioclimate variables used) to 1.0 (high stability, where a single variable is chosen each time). These summary statistics indicate 1) how variable is the predicted maximum tree cover, and 2) how stable is the identity of the limiting factor.

### Impacts of Unmeasured Factors

Unmeasured factors are identified as deviations between the predicted maximum tree cover and the actual tree cover for a site [16]. A deviation that indicates the presence of an unmeasured limiting factor is defined here as a greater than 10% difference between predicted *B*_*max*,*measured*_ and actual tree cover. We calculated the mean and standard deviation of this deviation. To estimate what fraction of the landscape appears to be limited by one of the measured factors, we identified all sites that had a percent deviation of less than or equal to 10%. All other sites were considered limited by an unmeasured factor.

### Evidence of limiting factors

Since there is an upper bound to the tree cover (100%), we can use this to determine if there is evidence of system-wide and per-bioclimatic variable limitations. If a per-bioclimatic limiting factor model *LF*_*Vi*_ yields ranges in which the maximum possible tree cover is at 100%, this variable is not limiting across these ranges. Similarly, across all variables, if a site is predicted to have a *B*_*max*,*measured*_ of 100% tree cover, the site itself can be consider to be unconstrained by any of the measured factors.

We summarized the system-wide and per-forest type class relative area of the most limiting factor for the entire study area as well as locations that had less than a 10% difference between predicted *B*_*max*,*measured*_ and actual tree cover.

### Multiple versus single variable predictions of limiting factor

As mean temperature is a commonly used bioclimatic variable in univariate models to constrain plant responses [10], we calculated the difference between the limiting factors predicted by the mean temperature surface alone to the limiting factors derived from the multiple variables approach. The system-wide mean and standard deviation of the differences were calculated.

## Results

### Bioclimatic limitations

**Fig 3:**
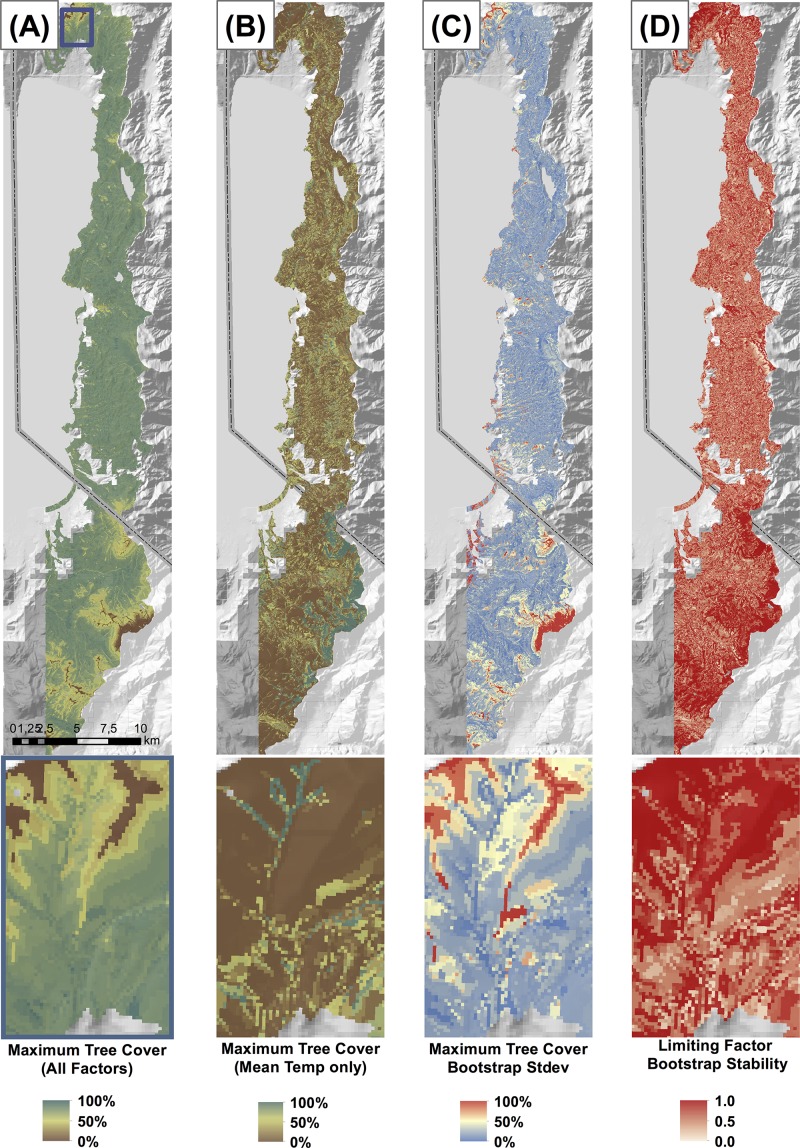
In panel a), Map of the predicted maximum tree cover for the study area under mean temperature, minimum temperature, potential evapotranspiration, and PET-P limitations. b) Map of the predicted maximum tree cover for the study under only mean temperature limitations. c) Map of the standard deviation of the all-factor maximum tree cover predictors generated from an N = 500 bootstrap. d) Map of the stability of the limiting factor identity predictions from an N = 500 bootstrap. Values of 1.0 indicate a single factor was chosen as the limiting factor in all bootstraps (most stable).

**Fig 4:**
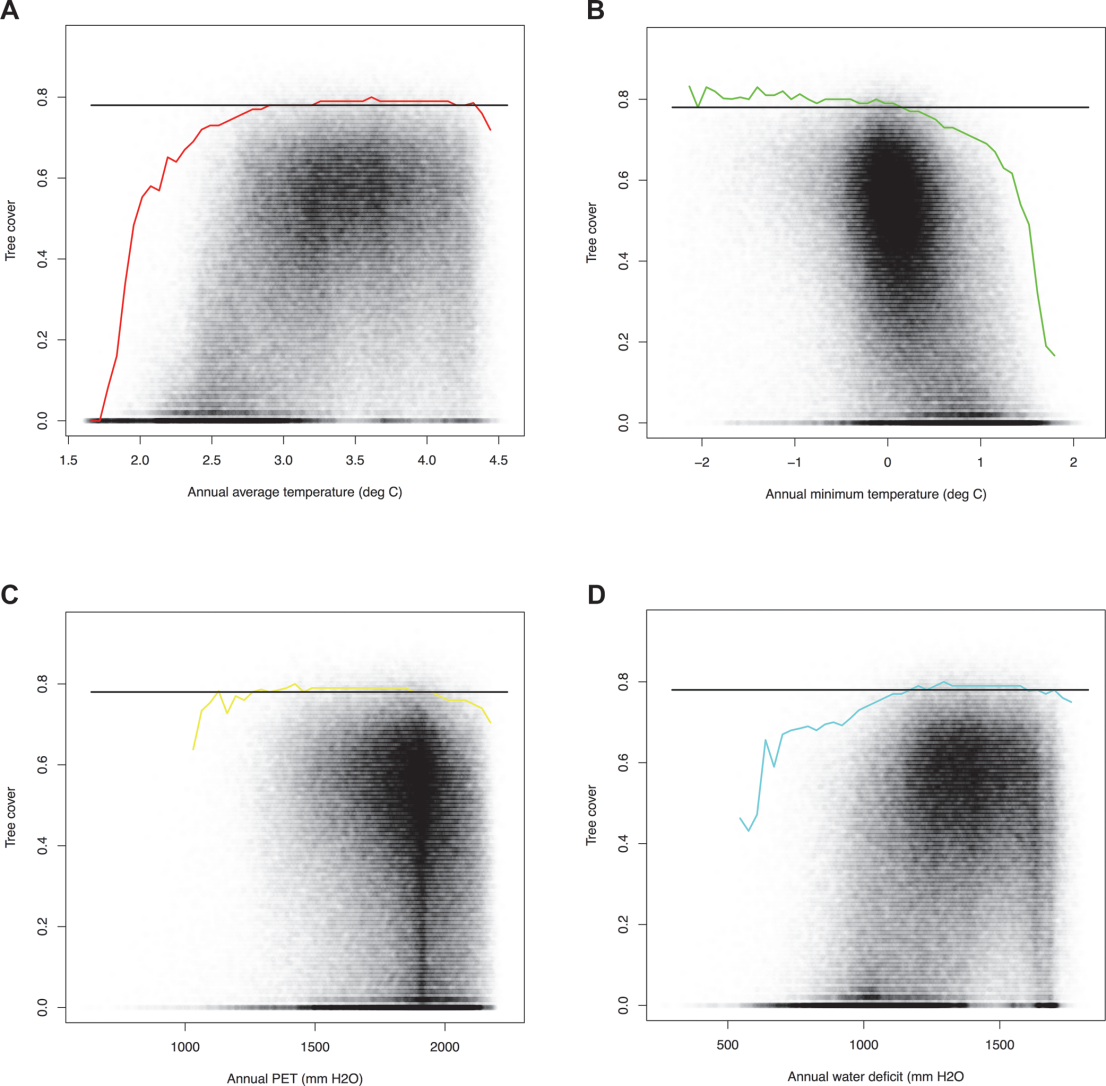
Tree cover (y axis) vs. bioclimatic variables. The scatterplot is density shaded. A-D. The colored line represents the 99% quantile (the environmental limiting factor) of that bioclimatic variable. The horizontal black line represents the study area 99% quantile of tree cover.

The actual tree cover for the study area averaged 39.7%, ranging from 0% to 94.1% (standard deviation +/- 22.2%). System-wide, the study area yielded a mean maximum tree cover of 86.3%, ranging from 5.8% to 91.8% with a standard deviation of +/- 6.1% (see Fig. 3A). All of the bioclimatic variables showed evidence of being limiting on tree cover, and all of the bioclimatic variables showed higher limiting effects at the tails of their ranges (Fig. 4, A-D). System-wide, the mean standard deviation of the predicted maximum tree cover across the N = 500 bootstraps was 0.009 (see Fig. 3C). The system-wide mean stability of the limiting factor prediction was 0.762 (possible range = 0.25 to 1.0), with a standard deviation of 0.196 (see Fig. 3D). The deviation between the predicted maximum tree cover and the actual maximum tree cover averaged 46.6%, with a standard deviation of +/- 22.2%. 1.2% of the landscape was found to have an actual tree cover be within 10% of the predicted maximum tree cover.

Based on the predicted maximum tree cover, 99.00% of the land area was predicted to be potential closed tree canopy forest, 0.65% open tree canopy forest, and 0.35% non-forest. By comparison, the actual classification of the landscape yielded 20.9% closed tree canopy forest, 51.5% open tree canopy forest, and 27.6% non-forest.

**Fig 5:**
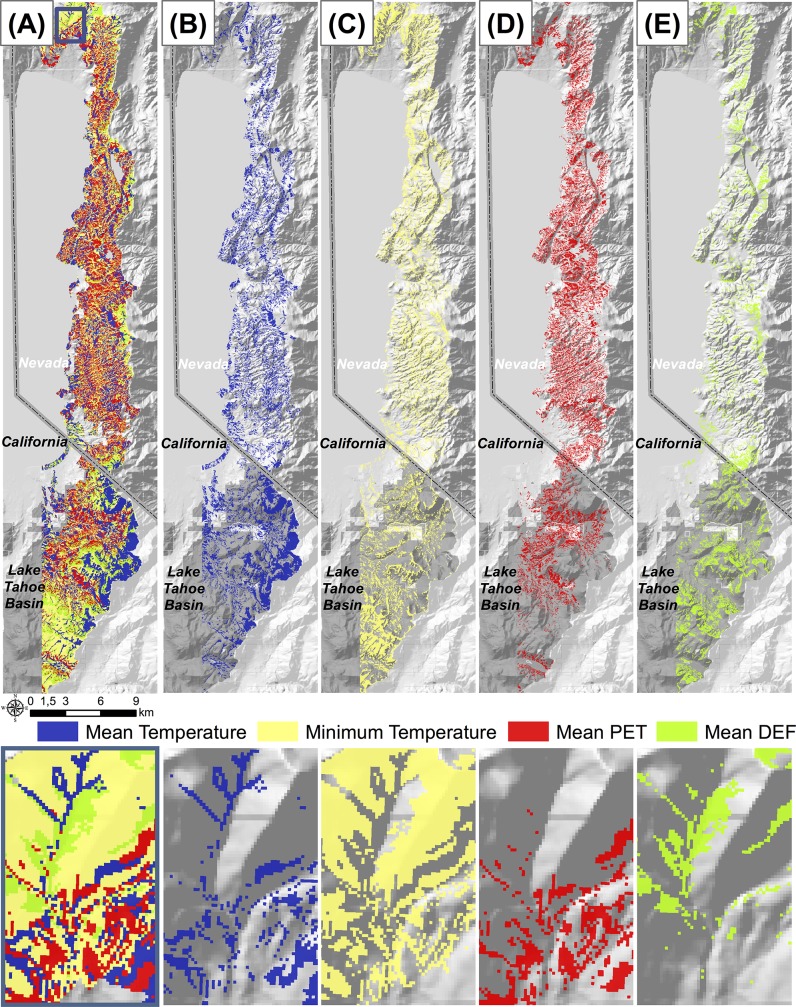
Map of which environmental limiting factors constrain maximum tree cover at a given location.

Of the measured factors, minimum temperature was the most common limiting factor across the study area, limiting 31.9% of the area. Mean temperature was the next most limiting (28.0%) followed by potential evapotranspiration (24.3%) and then PET-P (15.7%) (Fig. 5). Table 1 summarizes the relative contribution of each limiting factor overall, and broken down by potential forest type.

**Table 1:** Summary of total percent area constrained by each bioclimate variable for the entire study area as well as by forest type.

**Limiting factor**	**Overall**	**Closed Canopy Forest**	**Open Canopy Forest**	**Non-Forest**
Minimum temperature	31.9%	27.8%	39.5%	64.2%
Mean temperature	28.0%	31.9%	39.8%	29.7%
PET	24.3%	24.6%	5.3%	0.1%
PET-P	15.7%	15.8%	15.5%	6.0%

When only considering the fraction of the landscape with actual tree cover within 10% of the predicted maximum tree cover, we find the most limiting factor across the study area is PET (30.8%), followed by mean temperature (29.3%), minimum temperature (24.7%) and then PET-P (15.1%). Table 2 summarizes the relative contribution of each limiting factor broken down by potential forest type for all sites that are within 10% of the predicted maximum tree cover.

**Table 2:** Summary of the percent area constrained by each bioclimate variable for sites that are within 10% of the predicted maximum tree cover for the entire study area as well as by forest type.

**Limiting factor**	**Overall**	**Closed Canopy Forest**	**Open Canopy Forest**	**Non-Forest**
Minimum temperature	24.7%	25.2%	8.0%	3.4%
Mean temperature	29.4%	28.6%	12.0%	86.2%
PET	30.8%	31.2%	36.0%	0.0%
PET-P	15.1%	15.0%	44.0%	10.3%

The difference in the predicted maximum tree cover under the multiple-factor analysis (mean temperature, minimum temperature, PET and PET-P) vs. the single-factor analysis (mean temperature only) was 2.3% +/- 3.7%, ranging from 0% to 83.2% (see Fig. 3A vs. 3B). When broken down by potential forest types, closed canopy forests yielded a difference of 2.3% +/- 2.4%, open canopy forests 22.5% +/- 20.0%, and non-forests 45.3% +/- 31.1%.

## Discussion

Maximum tree cover was found to be limited across the entirety of our study area: no location was found to have 100% tree cover. All of the bioclimatic variables analyzed were found to limit tree cover to differing degrees. While the model indicated climate should result in a mean potential canopy cover of 86.3%, the actual measured canopy cover for the study area was 39.7%, with 1.2% of the sites falling within 10% of their potential maximum. Thus, much of the tree cover in the study area is controlled by factors that are note accounted for here. Where sites are within 10% of their predicted maximum, we found expected patterns of limitations: non-forests were dominated by cold mean temperature limitations, open-canopy forests were dominated by evaporative demand (PET and PET-P) limitations, and closed canopy forests, the least limited of the three forest types, showed no clear pattern of one factor being dominant over another.

An important difference between the analysis of ELFs and central-tendency approaches to understanding the relationship between climate and biological responses is the ability to clearly identify when and where unmeasured limiting factors are present, and to allow for new hypotheses to be generated. In this case, we found that very little of the area was limited by measured factors which are commonly used in plant-climate relationships, namely temperature, energy availability, and evaporative demand. We propose two potential limiting factors that may explain much of the discrepancy between the climate-predicted potential tree cover and the actual tree cover found within our study area: 1) disturbance history and 2) more accurate accounting of plant water availability based on soil characteristics.

Disturbance history has a clear link with potential tree cover: if a deforestation event (logging, fire, disease) were to have occurred, a site may not be at its theoretical maximum tree cover because there may not have been enough time that had elapsed between the disturbance event and when the tree cover could approach its theoretical maximum. The Lake Tahoe Basin experienced significant clear-cutting in the 19^th^ century [36]. Contemporary forests in the Basin, particularly the Jeffrey Pine forests that dominate the eastern side of the Basin, have been found to have significantly smaller trees than pre-settlement forests [36], supporting the notion that there simply has not been enough time for larger trees, with their corresponding large crown diameters and cover, to dominate the landscape.

Another potential missing factor is a more accurate representation of plant available water. Our results hint towards this being a major factor given the counter-intuitive findings that sites with high minimum temperatures resulted in extremely low tree cover, despite these conditions often being equated to beneficial to plant establishment and growth, particularly in more energy limited systems [26]. The mechanistic interpretation of these results may be due to an important, but unresolved, bioclimatic variable highly collinear with the bioclimatic variables we analyzed: plant available water (i.e. soil moisture). Visual examination of these sites showed large amounts of exposed bedrock, strengthening this interpretation. A preliminary analysis showed little correlation with polygon-based measures of soil depth and water holding capacity, but these datasets were too coarse to be used in this analysis. More realistic surfaces of soil properties (e.g. [30]) and soil water derived from a hydrological model would be needed to disentangle the effects of low soil water from the other collinear variables.

This analysis demonstrates several important principles: first, the analysis of ecological limiting factor can allow for the implementation of the principle of limiting factors to decouple highly correlated bioclimatic conditions, demonstrated in our study area by the high degree of stability in the limiting factor identity prediction. Despite the interrelationship between the four climate variables analyzed (average temperature, PET and PET-P all use minimum temperature in their calculations), the prediction of which variable was limiting where makes sense in light of previous findings in the Basin (e.g. [21]). Secondly, the use of a complete geospatial solution to fulfill the requirement of high sample sizes can be applied to other systems, scales and biophysical parameters, given the wealth of remotely sensed vegetation data. We show an application of the methodology to detect ELFs on tree cover data, which can be expanded to global tree cover products (e.g. [20]). Further, it can be extended to understand limitations on canopy density as measured by Leaf-Area Index remote sensing products [31]. From an ecosystem modeling standpoint, the use of tree cover (as opposed to discrete representations of land cover classes) results in models that are less sensitive to scaling effects [32]. Many regional or global scale models operate on Plant Functional Types (PFTs), not individual species [32]. This analysis can then provide key information for dynamic vegetation models [10], which require parameterization of climate tolerances of PFTs [33], and has the potential to extend these modeling approaches to include other ecosystem functional parameters such as productivity [34]. Beyond tree cover as a density measure, this methodology can be applied to abundance, and biomass surfaces. Finally, these approaches allow us to move away from the treatment of limiting factors in ecology as largely untestable models.
